# Prevalence and predictors of intimate partner violence among infertile women in a secondary health facility in South-South Nigeria

**DOI:** 10.4314/gmj.v59i3.8

**Published:** 2025-09

**Authors:** Ngozi R Maduka, Oseihie I Iribhogbe

**Affiliations:** 1 Department of Obstetrics and Gynaecology, Central Hospital, Agbor, Delta State, Nigeria; 2 Department of Obstetrics and Gynaecology, University of Benin Teaching Hospital, Benin City

**Keywords:** Intimate partner violence, Infertile women, South-south, Secondary Health Facility, Nigeria

## Abstract

**Objectives:**

To determine the prevalence of Intimate partner violence (IPV) in a secondary health facility,

**Design:**

A cross-sectional study

**Setting:**

Antenatal clinic of a secondary Health facility in Delta state, Nigeria, between July 1st, 2023, and June 30^th^, 2024

**Participants:**

244 women presenting for fertility management within the period of the study

**Main outcome measures:**

The proportion of women who suffered IPV due to infertility

**Results:**

The prevalence of IPV was 44.7%. Factors that were significantly associated with IPV were alcohol intake by the male partner [OR=2.74, 95% CI (1.25 - 6.01)], and male partner smoking habit [OR=5.90, 95% CI (2.50 – 14.00)]. Women in monogamous relationships were less likely to experience IPV [OR=0.12, 95% CI (0.02 - 0.60)]. Additionally, women who had given birth to one or more children with their partner were also less likely to suffer from IPV [OR=0.24, 95% CI (0.10 - 0.54)]. Psychological 40(16.4%), verbal 35(14.3%), and multiple 28 (11.5%) abuses were the most common forms of IPV. The majority of the participants, 110 (45.1%), would keep IPV a secret, while 54(22.1%) and 39(16.0%) would report to the family or resort to prayer.

**Conclusions:**

Overall, the study underscores the urgent need for targeted interventions addressing male substance abuse and societal attitudes towards infertility, particularly in polygamous households, to mitigate IPV against infertile women. Women should be educated on the importance of speaking up in the event of IPV.

**Funding:**

None declared

## Introduction

According to the World Health Organisation (WHO), infertility is a disease of the male or female reproductive system defined by the failure to achieve a pregnancy after 12 months or more of regular unprotected sexual inter-course.^1^ It is a global problem with regional variation in the prevalence rate, and the WHO estimates that 8–12% of couples would experience difficulty in conception after 1 year of regular unprotected sexual intercourse.^2^,^3^ Both male and female factors have been implicated in the aetiology of infertility, with each factor being responsible in about 30–40% of cases. In comparison, the remaining 30% are due to the interaction of both female and male factors.^4^ However, the woman is often blamed for the couple's infertility in most cases and suffers deprivation economically, socially, and emotionally as a consequence.^3^,^5^,^6^

Intimate partner violence (IPV) is a widespread and complex issue affecting millions of individuals worldwide.^7^ Violence against women, especially IPV, has been recognised as one of the most serious social problems in every society and is considered a violation of human rights. WHO defines IPV as any behaviour within an intimate relationship by an intimate partner that causes physical, psychological, or sexual harm to those in the relationship.^8^,^9^ It is one of the most common types of violence experienced by women.^8^,^9^ It is a multifaceted issue, influenced by a range of factors, including cultural norms, economic conditions, and social structures.^10^,^11^

Globally, over a third (35%) of women have experienced physical and/or sexual violence by an intimate partner or sexual violence by a non-partner at some point in their lives.^12^,^13^

Reports from the Nigerian national population commission estimated women's lifetime exposure to IPV from their current husband or partner at 19% for emotional IPV, 14% for physical IPV, and 5% for sexual IPV. 13 Infertile women were more likely to suffer domestic violence.^13^ Eka et al^14^ reported that the prevalence of violence among the infertile respondents was comparatively higher than among their fertile counterparts (62.5% and 54.2%, respectively) in their study in Makurdi. Sharifi et al in a recent meta-analysis showed that the prevalence of IPV against infertile women stood at 47.2% (95% CI: 34.660-59.850). Psychological and emotional violence were among the most common types of violence.^15^ Ameh et al.^16^ in Nigeria reported that 41.6% of infertile women experience marital violence as a result of infertility. Aduloju et al^17^ and Fiebai et al^18^ reported 31.2% and 32.6% prevalence of IPV among infertile women in Ado Ekiti and Port Harcourt, Nigeria, respectively.

Despite the prevalence of violence against women, research on IPV among infertile women in South-South Nigeria is limited. Given the region's unique cultural dynamics and their impact on IPV, this study aims to investigate the prevalence and predictive factors of IPV among women seeking infertility care at Central Hospital Agbor, Delta State, Nigeria. By exploring the prevalence and determinants of intimate partner violence among infertile women in this region, this study aims to enhance understanding of this pressing issue and guide initiatives to improve the health, well-being, and safety of this vulnerable group.

## Methods

**Study setting:** The study was conducted at the Central Hospital Agbor (CHA), Delta State, Nigeria. CHA is a secondary healthcare facility that was established in 1906. It is a 250-bed hospital located in the South-South region of Nigeria. It provides general medical care and specialist services to indigenes of Delta State and neighbouring parts of Edo State. The Obstetrics and Gynaecology department is led by two consultants who are fellows of the National Postgraduate Medical College of Nigeria and the West African College of Surgeons. Agbor is a kingdom in Delta State, Nigeria, occupying a part of Delta State that shares a boundary with Edo State. The people of Agbor town are Ika, and they speak the Ika dialect of the Igbo language. Agbor has a population of approximately 67,000 people, who are predominantly Christians of various denominations. Some of the indigenes practice African traditional religion, and there are a few migrant Hausa/Fulani Muslims. The primary occupations of the indigenes of Agbor are farming and trading.

**Study design:** This cross-sectional study was conducted at the fertility clinic of a Secondary Health facility in Delta state, Nigeria, between July 1st, 2023, and June 30^th^, 2024

**Inclusion and Exclusion criteria**: All patients who presented at the Gynaecology clinic during the study period with a diagnosis of infertility were recruited after providing informed consent

**Ethical clearance:** The study protocol was approved by the Central Hospital Agbor Ethical Committee with ethical number E/Com.AMZ/07/23. Recruitment of study participants was voluntary, based on verbal briefings in English, pidgin English, or the local languages, as appropriate. The information collected was entered and kept in a password-protected computer, maintaining the confidentiality of patients' records. The study was conducted in accordance with the guidelines of the Declaration of Helsinki (2013). The authors are available and ready to supply the data on request.

**Data collection**: The study's objectives were clearly explained to the women who met the inclusion criteria, and informed consent was obtained from them. A total of 244 women attending the infertility clinic who consented to participate in the study were consecutively recruited between July 1, 2023, and June 30, 2024. The instrument for data collection was a structured questionnaire developed from a previous study by Fiebai et al^18^ with some modifications. A total of 256 questionnaires were administered in the form of an interview by a trained clinic staff. The clinic staff ticked the corresponding answer on the questionnaire that the respondents answered. This method helped ensure that correct answers were entered and also reduced the attrition rate. Sociodemographic characteristics, fertility history, and details of IPV (Physical, verbal, psychological, and sexual violence) and Participants' Suggested Strategies for addressing IPV perpetrators were documented.

**Data analysis**: The data generated was analysed using the IBM Statistical Package for the Social Sciences (SPSS) version 25. Variables were presented with descriptive statistics, including frequencies, percentages, averages, and standard deviations, and were displayed in tables. Tests of significance for differences were performed at p values < 0.05 using the Chi-square test or Fisher's exact test, as appropriate for categorical variables. Logistic regression analysis was performed to determine the significant variables associated with IPV.

## Results

Of the 256 Questionnaires deployed for the study, only 244 of the returned questionnaires had complete information, and these were included in the analysis. The mean age of participants was 34.13 ± 5.67 years, with a minimum age of 22 years and a maximum age of 45 years. Age groups of 31-35 years and 36-40 years contributed 29.9% and 33.6%, respectively. As expected, participants were predominantly Christian, accounting for 99.2%, while Muslims made up only 0.8%. All participants had attained a formal education with secondary and tertiary levels contributing 49.2% and 44.3% for the participants and 45.9% and 51.6% for the male partners, respectively.

Owners of medium- and small-scale businesses comprised the majority, contributing 50.4% of the participants and their male partners, respectively. Alcohol and smoke consumption among the male partners were 73.0% and 18.9%, respectively. (Table 1)

**Table 1:** Sociodemographic characteristics of participant S (N=244)

Characteristics	Frequency, n(%)
**Age**	
**21-25**	12(4.9)
**26-30**	49(20.1)
**31-35**	73(29.9)
**36-40**	80(33.6)
≥**41**	28(11.5)
**Religion**	
**Catholics**	40(16.4)
**Anglicans**	6(2.5)
**Pentecostals**	196(80.3)
**Moslems**	2(0.8)
**Tribe**	
**Ika**	113(46.3)
**Igbos**	83(33.6)
**Urhobos**	5(2.0)
**Others**	44(18.0)
**Wife Education**	
**Primary**	16(6.6)
**Secondary**	120(49.2)
**Tertiary**	108(44.3)
**Wife Occupation**	
**Civil servant**	30(12.3)
**Traders**	123(50.4)
**House managers**	17(7.0)
**Professionals**	18(7.4)
**Artisans**	56(23.0)
**Husband Education**	
**None**	3(1.2)
**Primary**	3(1.2)
**Secondary**	112(45.9)
**Tertiary**	126(51.6)
**Husband occupation**	
**Civil servant**	31(12.7)
**Traders**	90(36.9)
**Professionals**	20(8.2)
**Artisans**	103(42.2)
**Alcohol habit**	
**Consume**	178(73.0)
**Don't consume**	66(27.0)
**Smoking Habit**	
**Smoke**	46(18.9)
**Don't smoke**	198(81.1)

The majority of participants, 230 (94.3%), came from monogamous families, with 203 (83.2%) legally married to their partners. Secondary infertility, affecting 212 participants (86.9%), was the predominant type of infertility, while 121 participants (49.6%) had been pregnant for their partners before. Only 59 participants (24.2%) had one or more children with their partners. Family upkeep was a shared responsibility among 189 participants (77.5%), while only 2 participants (0.8%) admitted to being solely responsible for it. Women who experienced infertility lasting 5 years or more comprised the majority, 106 participants (43.4%). (Table 2)

**Table 2:** Fertility History and Marital Status of Respondents

Characteristics	Frequency, n(%)
**Type of marriage**	
**Monogamy**	230(94.3)
**Polygamy**	14(5.7)
**Legality of marriage**	
**Legal**	203(83.2)
**Not legal**	41(16.8)
**Type of Infertility**	
**Primary**	32(13.1)
**Secondary**	212(86.9)
**Pregnant before for partner**	
**Yes**	121(49.6)
**No**	123(50.4)
**Any child for partner**	
**Yes**	59(24.2)
**No**	185(75.8)
**Family upkeep**	
**Husband**	53(24.2)
**Wife**	2(0.8)
**Both**	189(77.5)
**Duration of Infertility**	
**≤2 years**	72(29.5)
**3-4 years**	66(27.0)
**≥5 years**	106(43.4)

The prevalence of IPV was 44.7 %. Among the 244 participants, 109 (55.3%) reported suffering one or multiple forms of abuse. The most prevalent form experienced by the respondents was psychological abuse, affecting 40 (16.4%) of the total population. Additionally, 35 (14.3%) of them reported experiencing verbal abuse, while multiple abuse, sexual, and physical abuses were reported by 28 (11.5%), 4(1.6%), and 2 (0.8%), respectively.

Sociodemographic characteristics affecting the prevalence of IPV were the husband's occupation (p<0.05), alcohol intake by the male partner (p <0.05), and smoking habit of the male partner (p<0.05) (Table 3)

**Table 3:** Association between IPV and Sociodemographic characteristics

Characteristics	Abused n=109 (%)	Not Abused n=135 (%)	P value
**Age**			
**21-25**	3 (1.3)	9(3.7)	
**26-30**	22 (9.0)	27(11.1)	
**31-35**	25(10.3)	48(19.7)	0.064
**36-40**	43(17.6)	37(15.2)	
**≥41**	14()5.7	14(5.7)	
**Religion**			
**Catholics**	17 (7.0)	23(9.4)	
**Anglicans**	3 (1.2)	3(1.2)	
**Pentecostals**	89(36.5)	107(43.9)	0.614
**Moslems**	0 (0.0)	2(0.8)	
**Tribe**			
**Ika**	53(21.7)	60(24.6)	
**Igbos**	33(13.5)	50(20.5)	
**Urhobos**	3(1.2)	2(0.8)	0.588
**Others**	21(8.6)	23(9.4)	
**Wife Education**			
**Primary**	10(4.1)	6(2.5)	
**Secondary**	53(21.7)	67(27.5)	0.323
**Tertiary**	46(18.9)	62(25.4)	
**Wife Occupation**			
**Civil servant**	14(5.7)	16(6.6)	
**Traders**	50(20.5)	73(29.9)	
**House managers**	8(3.3)	9(3.7)	0.165
**Professionals**	5(2.1)	13(5.3)	
**Artisans**	32(13.1)	24(9.8)	
**Husband Education**			
**None**	2(0.8)	1(0.4)	
**Primary**	2 (0.8)	1(0.4)	0.450
**Secondary**	54(22.1)	58(23.8)	
**Tertiary**	51(21.0)	75(30.7)	
**Husband occupation**			
**Civil servant**	11(4.5)	20(8.2)	
**Traders**	50(20.5)	40(16.4)	0.002*
**Professionals**	2(0.8)	18(7.4)	
**Artisans**	46(18.9)	57(23.4)	
**Alcohol habit**			
**Consume**	94(38.5)	84(34.4)	0.001*
**Don't consume**	15(6.2)	51(20.9)	
**Smoking Habit**			
**Smoke**	37(15.2)	9(3.7)	0.001*
**Don't smoke**	72 (29.5)	126(51.6)	

* Significant

Two key marital factors were found to influence the likelihood of IPV, the type of marriage and the presence of children. Specifically, women in polygamous relationships were at a significantly higher risk of experiencing IPV (p<0.05), while women without children with their partner were also more likely to experience IPV (p<0.05) (Table 4)

**Table 4:** Association between IPV and marital status

Characteristics	Abused n=109(%)	Not Abused n=135(%)	P value
**Type of marriage**			
**Monogamy**	97((39.8)	133(54.5)	0.02*
**Polygamy**	12(4.9)	2(0.8)	
**Legality of marriage**			
**Legal**	93(38.1)	110(45.1)	0.267
**Not legal**	16(6.6)	25(10.3)	
**Type of Infertility**			
**Primary**	12(4.9)	20(8.2)	0.248
**Secondary**	97(39.7)	115(47.1)	
**Pregnant before for partner**			
**Yes**	49(20.1)	72(29.5)	0.120
**No**	60(24.6)	63(25.8)	
**Any child for partner**			
**Yes**	16(6.6)	43(17.6)	0.01*
**No**	93(38.1)	92(37.7)	
**Family upkeep**			
**Husband**	23(9.4)	30(12.3)	
**Wife**	2(0.8)	0 (0.0)	0.284
**Both**	84(34.4)	105(43.0)	
**Duration of Infertility**			
**≤2 years**	27(11.1)	45(18.4)	
**3-4 years**	26(10.7)	40(16.4)	0.078
**≥5 years**	56(23.0)	50(20.5)	

* Statistically significant

From the logistic regression analysis (Table 5), women married to men who consume alcohol were more likely to experience abuse [OR=2.74, 95% CI (1.25 - 6.01)]. Furthermore, the smoking habit of the male partner significantly increased the likelihood of IPV [OR= 5.90, 95% CI (2.50 - 14.00)]. Conversely, women in monogamous relationships were less likely to experience IPV [OR=0.12, 95% CI (0.02 - 0.60)]. Additionally, women who had given birth to one or more children with their partner were also less likely to suffer IPV [OR=0.24, 95% CI (0.10 - 0.54)].

**Table 5:** Logistic regression of the risk factors associated with the experience of IPV as the dependent variable

Variable	UOR (95% CI for UOR)	P value	AOR (95% CI for AOR)	P value
**Husband occupation**				
**Civil servants**	1.47(0.64-3.37)	0.366	1.37(0.52-3.59)	0.520
**Traders**	0.65(0.36-1.14)	0.132	1.91(0.95-3.84)	0.069
**Professionals**	7.26(1.60-32.93)	0.10	0.21(0.33-1.31)	0.095
**Artisans^†^**				
**Alcohol habit**				
**Consume**	3.80(1.99-7.26)	0.001*	2.74(1.25-6.01)	0.012*
**Don't consume**				
**Smoking habit**				
**Smoke**	7.19(3.29-15.76)	0.001*	5.90(2.50-14.00)	0.001*
**Don't smoke**				
**Type of Marriage**				
**Monogamy**	0.12(0.03-0.56)	0.001*	0.12(0.02-0.60)	0.010*
**Polygamy**				
**Any child for the partner**				
**Yes**	0.37(0.19-0.70)	0.02*	0.24(0.10-0.54)	0.001*
**No**				

† Reference Category

* Statistically significant

When participants were asked how they would respond to partner abuse, nearly half (45.1%) of the participants said they would keep it to themselves, while 54 (22.1%) and 39(16.0%) planned to seek help from family members or turn to prayer, respectively. Multiple actions were suggested by 24 (9.8%) of participants, and 6 (2.5%) mentioned that they would pursue legal action. (Table 6)

**Table 6:** Participants' Suggested Strategies for addressing IPV perpetrators

Attitude	Frequency (244)	Percentage (%)
**Keep it to myself**	110	45.1
**Report to family**	54	22.1
**Resort to prayers**	39	16.0
**Multiple actions**	24	9.8
**Legal redress**	6	2.5
**Report to the church**	6	2.5
**Inform Doctor**	5	2.0

## Discussion

From the study, the prevalence of IPV among infertile women was 44.7%. This rate is higher than the reported prevalence among the general population. The 2018 Nigerian National Demographic Survey indicated a prevalence of 33.8% of IPV among the general population. Oseni et al. (20) reported a prevalence of IPV of 37.7% in the general population of the three senatorial districts of Edo State, South-South Nigeria, in their survey. This study revealed a high rate of IPV among infertile women, indicating a significant burden of IPV in this population. Notably, this high prevalence exists despite the fact that infertility often results from combined factors involving both male and female partners, highlighting a concerning disparity in the experiences of infertile women. When compared to other studies on IPV among infertile women, the prevalence was slightly higher than the findings by Ameh et al^16^ who reported a prevalence of 41.6% in a multicentre study comprising Zaria, Abuja, and Abakiliki. Aduloju et al 17 in Ekiti, Nigeria, and Fiebai et al 18 in Port Harcourt, Nigeria reported prevalence of 31.2% and 32.3%, respectively. In a recent study by Ashimi et al^21^ where participants were recruited from three geopolitical zones of Nigeria, the prevalence of IPV among infertile women was 39.8%. In Egypt and Nepal, Ahmed et al. 22 and Silwal et al. 23 reported prevalences of 50.5% and 55.4%, respectively. Lower values of 15.0% have been reported in a study by Sahin et al^25^ in Turkey. The wide variation reported in the various studies may be a reflection of cultural differences in the study population and the premium placed on childbirth.

The community/cultural perception of Domestic violence could also play a role, as many cases of domestic violence go unreported due to cultural beliefs. For example, among the TIV-speaking people of Nigeria, it was reported that wife beating is even regarded as a sign of love, which the women have been socialised to accept and even encourage.^25^ Violence against women by male partners is also widely condoned by many Nigerian societies, where the belief that a husband may chastise his wife by beating her is deeply embedded in the culture.^26^

After accounting for potential confounding variables, four key factors were identified as significant predictors of IPV: the male partner's alcohol and smoking habits, the type of family system (monogamous or polygamous), and the partner's previous history of childbirth. Specifically, women partnered with men who use alcohol and tobacco are at increased risk of IPV. Alcohol consumption can impair judgment and increase aggression, making it more likely for individuals to engage in violent behaviour. Smoking and alcohol use are also often linked to stress and coping mechanisms that might contribute to a hostile domestic environment. Furthermore, individuals who regularly consume alcohol and smoke may also be more prone to social circles and environments where aggressive behaviours are normalised, further increasing the risk of IPV. Additionally, polygamous relationships and childlessness were associated with higher IPV rates. In contrast, the history of previous childbirth was linked to lower IPV incidence. In polygamous contexts, the dynamics among co-wives can engender feelings of jealousy and competition, potentially leading to heightened tension and conflict within the household. To manage these complex interactions, husbands may resort to control mechanisms or show favouritism, which can sometimes escalate into violence.

In our cultures where childbearing is highly valued, women who are unable to conceive may find themselves at a disadvantage, as societal expectations and traditional norms often place significant pressure on fertility. This situation may be exploited by partners through emotional mistreatment or belittlement, thereby contributing to IPV. Moreover, male partners might perceive childlessness as a failure to meet established marital expectations, which could become a justification for abusive behaviour and attempts to exert control within the relationship. This issue invites further exploration into the intersection of polygamy, societal norms around fertility, and the dynamics of IPV.

The most common type of abuse suffered by infertile women in this study was psychological abuse (36.7%), while 32.1% of the abused women reported having suffered verbal abuse. Although the value was relatively lower, the finding is similar to the study by Ameh et al^16^ and Adoluju et al^17^, who reported 51.5% and between 54.4-62.4% psychological abuse as the most common type of IPV in their study. The previous studies,^16^,^17^, however, did not take into consideration women who suffered multiple abuses. Among the women who reported multiple abuse, psychological abuse was one of the major components of their abuse. It is also possible that some of the participants may have come to terms with psychological abuse and no longer report it as a form of abuse. Despite the lower reported rates of physical and sexual violence in this study compared to other previous studies,^16^,^17^,^18^,^21^, it's likely that women underreported these incidents due to shame and cultural norms that physical violence may not be viewed as a form of violence. There could also be a decline in sexual interest from partners of IPV patients after an infertility diagnosis, potentially resulting in an underestimation of the actual prevalence of these forms of violence.

Women experiencing domestic violence have varying responses and differences in who they report their abuses to. The majority of the participants (45.1%) in this study resolved to keep issues of IPV a secret. This is similar to the findings in the Nigerian Demographic and Health Survey 2013, which reported that 45% of women who experienced violence never sought help or never told anyone about the violence.^27^

This may be due to fear of reprisals from their husbands, desire to protect their marriage, ridicule from family members and friends, and religious considerations.^28^ This may also explain why 16.0% of the participants opted for prayers rather than disclose the occurrence of IPV. Culture and tradition play a significant role in women's and community perceptions of IPV, which is reflected in the various approaches suggested by women for addressing IPV. The family system remains a very important channel for the settlement of disputes, including marital problems; hence, the response by 22.1% of the participants that suggested that IPV should be reported to the families is not surprising. The very low response rate (2.5%), which suggests that IPV should be addressed through legal means, may reflect the respondents' distrust in the legal system. It is regarded as taboo to involve the police in family matters, and this has also contributed to the high incidence of underreporting.^29^ When victims report to their families, there's a risk that the issue may be concealed from public attention, either due to fear of retaliation from the abuser or lack of trust in law enforcement, which often views domestic violence as a private matter. Although domestic violence violates fundamental human rights, which the Nigerian Constitution opposes, certain laws, such as the Penal Code in Northern Nigeria, perpetuate gender-based violence by permitting wife-beating under the guise of “correction” (Section 55 (1) (d).^30^ Despite these challenges, it is crucial to educate victims of IPV to report cases to the relevant authorities for official documentation, investigation, and potential justice.

Although Nigeria ratified the Convention on the Elimination of All Forms of Discrimination Against Women (CEDAW) in 1985, the treaty's effectiveness is hindered by the requirement that Parliament must enact corresponding domestic legislation to bring it into force; otherwise, the international agreement will remain largely unimplemented and ineffective.

This study was conducted in a semi-urban community comprising women from different socioeconomic strata. It therefore had a fair representation of various groups of women seeking fertility care and their experiences with IPV. However, being a facility-based study, the findings cannot be generalised to represent the entire population. Many of the women who suffered IPV in the past may not be able to recall the event, while some may have refused to disclose. Therefore, the problem of recall bias and nondisclosure cannot be completely ruled out, and as such, we presume that the true prevalence may even be higher than reported. Data collection was done by trained staff (a health assistant and a senior medical record officer) who were not directly involved in the patients' management. However, since they also work in the facility, there is a possibility of some biases or false information.

IPV screening, counselling, and referral services should be integrated into infertility treatment to help promote safer and more supportive relationships for affected couples. Furthermore, public awareness campaigns and education programs are recommended to help increase disclosure among affected women.

## Conclusion

This study reveals a significant prevalence of IPV among infertile couples in Agbor and its environs, with psychological, verbal, and multiple abuses being the most common experiences. The findings highlight the complex interplay between infertility, cultural norms, and relationship dynamics, which can exacerbate violence. The study's results underscore the need for healthcare providers, policymakers, and community leaders to address IPV as a critical aspect of infertility care and support.
